# *Influenza A* Virus Pre-Infection Exacerbates *Pseudomonas aeruginosa*-Mediated Lung Damage Through Increased MMP-9 Expression, Decreased Elafin Production and Tissue Resilience

**DOI:** 10.3389/fimmu.2020.00117

**Published:** 2020-02-13

**Authors:** Berengère Villeret, Brigitte Solhonne, Marjolène Straube, Flora Lemaire, Aurélie Cazes, Ignacio Garcia-Verdugo, Jean-Michel Sallenave

**Affiliations:** ^1^Inserm, UMR1152, Laboratoire d'Excellence Inflamex, Département Hospitalo-Universtaire FIRE (Fibrosis, Inflammation and Remodeling), Université de Paris, Paris, France; ^2^Assistance Publique-Hôpitaux de Paris (APHP), Hôpital Bichat, Service de Pneumologie A, Paris, France

**Keywords:** *Pseudomonas aeruginosa*, *influenza* virus, elafin, metalloprotease, lung tissue resilience

## Abstract

Individuals with impaired immune responses, such as ventilated and cystic fibrosis patients are often infected with *Pseudomonas aeruginosa* (*P.a*) bacteria, and a co-infection with the *Influenza* virus (IAV) is often present. It has been known for many years that infection with IAV predisposes the host to secondary bacterial infections (such as *Streptococcus pneumoniae* or *Staphylococcus aureus*), and there is an abundance of mechanistic studies, including those studying the role of desensitization of TLR signaling, type I IFN- mediated impairment of neutrophil chemokines and antimicrobial production, attenuation of IL1β production etc., showing this. However, little is known about the mechanistic events underlying the potential deleterious synergy between *Influenza* and *P.a* co-infections. We demonstrate here *in vitro* in epithelial cells and *in vivo* in three independent models (two involving mice given IAV +/– *P.a*, and one involving mice given IAV +/– IL-1β) that IAV promotes secondary *P.a*-mediated lung disease or augmented IL-1β-mediated inflammation. We show that IAV-*P.a*-mediated deleterious responses includes increased matrix metalloprotease (MMP) activity, and MMP-9 in particular, and that the use of the MMP inhibitor improves lung resilience. Furthermore, we show that IAV post-transcriptionally inhibits the antimicrobial/anti-protease molecule elafin/trappin-2, which we have shown previously to be anti-inflammatory and to protect the host against maladaptive neutrophilic inflammation in *P.a* infections. Our work highlights the capacity of IAV to promote further *P.a*-mediated lung damage, not necessarily through its interference with host resistance to the bacterium, but by down-regulating tissue resilience to lung inflammation instead. Our study therefore suggests that restoring tissue resilience in clinical settings where IAV/*P.a* co-exists could prove a fruitful strategy.

## Introduction

Individuals with impaired immune responses, such as cystic fibrosis (1–5) and ventilated patients (6, 7) demonstrate frequent respiratory viral infections with a variety of viruses, including the *Influenza A* virus (IAV). For example, clinical studies have shown correlations between viral infections with pulmonary exacerbations (1–3, 5), with the former often predisposing the host to secondary bacterial infections (8–11). Specifically, in a CF population study spanning over 6 years, which recruited in excess of 31,000 individuals and which reported an excess of 91,000 pulmonary exacerbations, the latter were associated with IAV activity (mostly of the H3N2 serotype) in both children and adults, with a *Pseudomonas aeruginosa* (*P.a)* prevalence of 48 and 82%, respectively. By contrast, an RSV association was only observed in adults (12). Surprisingly however, despite their simultaneous occurrence in such situations as acute nosocomial infections or chronic exacerbations in cystic fibrosis (see above) and COPD/emphysema, none of these studies have extensively tackled the interaction between *Influenza* and *Pseudomonas aeruginosa* (*P.a*). Indeed, mechanistic studies dealing with IAV and bacteria have mainly concerned *Staphylococcus aureus* and *Streptococcus pneumoniae*, for the latter, and a variety of mechanisms (often potentially contradictory) have been put forward to explain the deleterious consequences of such interactions.

These include, not exhaustively however, IAV-mediated desensitization of TLR signaling (13), type I interferon-mediated impairment of neutrophil chemokines or function (14–17), down-regulation of antimicrobial production (18–20), attenuation of IL1β production or IL1β-mediated alveolar macrophage activity (21, 22), exaggerated inflammatory responses (23, 24), or loss of lung repair potential (25, 26).

Mechanistically, we demonstrated here *in vitro* and *in vivo* in three independent models (two involving mice given IAV +/– *P.a*, and one involving mice given IAV +/– IL-1β) that IAV pre-treatment promoted secondary PAO1-mediated lung disease or augmented IL-1β-mediated inflammation, by enhancing deleterious inflammatory responses. These included increased matrix metalloprotease (MMP) activity, particularly MMP-9. Importantly, we showed that the MMP inhibitor batimastat improved lung resilience, and interestingly, this was not associated with an increase in bacterial clearance. Furthermore, we showed that IAV post-transcriptionally inhibited the antimicrobial/anti-protease molecule elafin/trappin-2, which we have shown previously to be anti-inflammatory in a variety of settings (27–29) and protects the host against maladaptive neutrophilic inflammation in *P.a* (27, 28, 30) infections, as well as against Plasmodium-mediated lung damage (29).

Altogether, out work highlights the capacity of IAV to promote further PAO1-mediated lung damage, not through its interference with host resistance to the bacterium (16, 19), but through down-regulating tissue resilience to lung inflammation instead. Our study therefore suggests that restoring tissue resilience in clinical settings where IAV/*P.a* co-exist could be a fruitful strategy.

## Materials and Methods

### Materials

Phosphoramidon and batimastat were obtained from Sigma-Aldrich. Recombinant human and murine interleukin 1β were purchased from R&D Systems. Tace II metalloprotease substrate was obtained from Enzolife Science. Neutrophil elastase (NE) was obtained from Elastin products.

### PAO1 and *Influenza A* Preparation

PAO1 WT (obtained from the ATCC; 15692), was grown overnight in Luria Broth (LB) medium (1% Bactotryptone, 0.5% Bacto Yeast Extract, 0.5% NaCl) under agitation. On the next day, an aliquot of PAO1 culture was grown over 3–4 h in an exponential phase and the OD was checked (600 nm). Bacteria were then centrifuged (4,000 rpm for 15 min) and pellets resuspended at the desired multiplicity of infection (moi) or colony forming units (cfu) in PBS.

The virus strain A/Scotland/20/74 (H3N2) was routinely amplified in MDCK cells (ATCC CCL-34), as described before Barbier et al. (31) and Villeret et al. (32). From the supernatants of infected cells, the *influenza* virus was purified by centrifugation in sucrose gradient, quantified by the virus plaque assay (32), aliquoted, and kept at −80°C until use.

When needed, purified samples were inactivated by heating at 95°C for 10 min in a block heater. Inability of heat-inactivated *influenza* virus (IAV^*^) to replicate was then confirmed by viral gene M2 q-PCR analysis (see below).

### Adenovirus Constructs

The replication-deficient adenovirus (Ad) Ad-MCMV-elafin is described in Sallenave et al. (33).

### Cells, Cell Cultures, and Protocols

NCI-H292 cells (ATCC reference number CRL-1848), a human pulmonary mucoepidermoid carcinoma cell line, BEAS-2B cells (ATCC CRL-9609), a SV-40 transformed bronchial epithelial cell line, and A549 (ATCC CCL-185) a cell line from a lung adenocarcinoma, were cultured in RPMI (NCI-H292) or F12/K Nutrient mixture (BEAS-2B and A549) medium supplemented with Glutamax, antibiotics, and 10% de-complemented fetal calf serum (all reagents from Gibco). Cells were incubated at 37°C in a water-jacketed CO_2_ incubator. Cells were infected in serum-free medium with either IAV or PAO1. Alternatively, they were stimulated with either h-IL-1β, 5′ triphosphate double stranded RNA (5′ ppp dsRNA at 1.2 μg/ml) (Invivogen), complexed to lipofectamine 2000 (Invitrogen), with polyinosinic-polycytidylic acid (poly IC at 10 μg/ml) (Invivogen), or with combinations thereof.

Cell viability was assessed by measuring Lactate dehydrogenase (LDH) activity in cell lysates and supernatants, using the CytoTox 96 Nonradioactive Cytotoxicity assay (Promega).

Cells were washed twice with ice-cold PBS and lysed in TrisHCl 50 mM, NaCl 150 mM, NP40 1%, Glycerol 3%, EDTA 2 mM, and EGTA 2 mM buffer. After centrifugation (14,000 rpm, 15 min, 4°C) pellets were discarded. Cell supernatants and lysates were then recovered and stored at −80°C until further analysis.

### *In vivo* Experiments

Procedures involving mice were approved by our Ethical Committee (Paris-Nord/No 121) and by the French ministry of Research (agreement numbers 4537.03 and 02012). Eight-week-old male C57Bl/6 mice and human elafin/trappin-2 transgenic mice (hereafter called eTg mice) were obtained from Janvier (Le Genest-Saint-Isle, France) and generated by our group (34), respectively. Mice were anesthetized using an intramuscular injection of ketamine 500 and xylazine 2% in 0.9% NaCl (20:10:70). Either the *Influenza A* virus (IAV), PAO1 bacteria, or m-IL-1ß recombinant protein were given intra-nasally (i.n) or through the oro-pharyngeal route, in a final volume of 40 μl instilled through a fine polypropylene tubing. Mice were then monitored for survival or were humanely killed (overdose of 100 μl intra-peritoneally-injected pentobarbital) for mechanistic studies. For the latter, bronchoalveolar lavages (BALs) fluid was obtained by cannulating the trachea and instilling 2 × 1 ml of PBS. Typically, a volume of 1.7 ml of BALF was retrieved and centrifuged at 2,000 rpm for 10 min. Supernatants were used for protein, cytokine/chemokine (ELISA), protease activity, and hemoglobin, as a surrogate for lung damage (absorbance reading at 405 nm) measurements. BAL cell pellets were used to perform cytospins for cell differential analysis (Diff-Quick, Dade Diagnostika GmbH, Unterschleissheim, Germany).

Lung tissues were used for RNA quantification, for assessment of bacterial count, after plating extracts on agarose plates, or for histological studies.

### Cytokines/Chemokines/Antimicrobials Measurement

The concentration of mediators in cell cultures supernatants/lysates or murine BALs were quantified by sandwich ELISA kits following the manufacturer's indications (R&D Bio-Techne, Minneapolis, MN) or used in our in house ELISA (33).

### BAL Protease Activities

BAL metalloprotease, trypsin-like, and neutrophil-elastase activities were measured using fluorogenic substrates, as described in Barbier et al. (31) Bastaert et al. (35), and Le Gars et al. (36), respectively. Alternatively, BAL MMP activity was measured by zymography (30).

### RNA Preparation

Cell monolayers were directly lysed in RNA lysis buffer. RNA isolation was performed using the PureLink® RNA Mini Kit (12183018A, Ambion, Life technologies), following the manufacturer's instructions. For lung RNA assessment, frozen lungs were homogenized in RNA lysis buffer provided by the Pure Link RNA extraction kit (Life Technologies), using lysing matrix D tubes and the FastPrep-24 5G mixer (MP Biomedical) at 4°C (two cycles of 40s, level 5). Briefly, lysates were mixed with 70% ethanol and loaded onto a silica-membrane column. After different washings, total RNA was eluted in DNAse-RNAse-free water and stored at −80°C until use.

DNase treatment was performed prior to Reverse transcription polymerase chain reaction (RT-PCR) using RNAse-free DNAse I (Roche) at 37°C for 10 min. DNAse was then inactivated by increasing the temperature to 70°C for 10 min. Complementary DNA (cDNA) was synthesized from total RNA (500 ng) using M-MLV Reverse Transcriptase (Promega) as per the supplier's protocol (1 h at 37°C followed by 1 min at 70°C).

Real-time PCR was done in a 7500 Fast Real-Time PCR System (Applied Biosystems). Reactions were performed in a total volume of 15 μl using 2x Fast SYBR® Green Master Mix (Life Technologies), 2 μl of diluted cDNA, 2 μmol forward primer, and 2 μmol reverse primer in a 96-well plate. PCR was run with the standard program: 95°C 10 min, 40 times of cycling 95°C 15 s and 60°C 1 min in a 96-well plate. Triplicate Ct values were obtained, and results were expressed as dCT = CT gene of interest-CT HPRT/18S (with low and high values representing high and low levels of the gene of interest, respectively). Alternatively, the results were expressed using the comparative Ct (ΔΔCt) calculation and the following formula: Fold change (*RQ*) = *2*^-(ΔΔ*CT*)^, using “control cells” as calibrator (arbitrary unit =1).

The primers used were the following: M2 viral protein: (Fw: aagaccaatcctgtcacctct; Rw: caaagcgtctacgctgcagtc); CCL-5: (Fw: cagtcgtctttgtcacccgaa; Rw: tcccaagctaggacaagagca); IL-8 (Fw: agagacagcagagcacacaa; Rw: ttagcactccttggcaaaac); IL-6 (Fw: tcaatgaggagacttgcctg; Rw: tgtactcatctgcacagcctc); HPRT (Fw: ttgctttccttggtcaggca; Rw: atccaacacttcgtggggtc); 18s rRNA (Fw: cttagagggacaagtggcg; Rw: acgctgagccagtcagtgta).

### Histology

Perfused and fixed lungs (4% PFA in PBS, overnight at 4°C) were embedded in paraffin and sectioned in slides (4 μm), stained with Hematoxylin-eosin. Inflammation was scored with a semi-quantitative scale (0: no inflammation to 4: severe inflammation with exudate) both in alveolar and in peribronchial/interstitial lung compartments.

### Statistical Analysis

Data were expressed as means ± standard errors of the mean (SEM) unless otherwise stated. One-way ANOVA was used to determine statistically significant differences among groups followed by Tukey's multiple test for comparisons. Survival curves in murine model experiments were plotted using Kaplan-Meier curves and statistical tests were performed using the Log-rank (Mantel-Cox) test. All analyses were performed with Prism version 7, GraphPad.

## Results

### IAV Pre-Infection Exacerbates *P. aeruginosa* Inflammation in C57Bl/6 Murine Lungs

**Figure 1:**
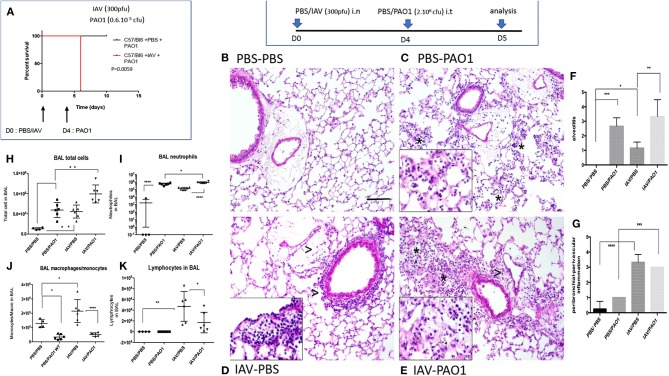
IAV lung pre-infection sensitizes WT C57Bl/6 mice to further PAO1-mediated inflammation. **(A)** Survival experiment: C57Bl/6 WT mice were instilled i.n with either PBS or IAV (300 pfu) (two cohorts of six animals for each treatment). Four days later, mice were further instilled i.t with PAO1 (0.6.10^6^ cfu), and mice survival was assessed, using Kaplan-Meier curves. Statistical tests were performed with the GraphPad Prism 6 package, using the Log-rank (Mantel-Cox) test, *p* = 0.0059. **(B–K)** Mechanistic experiment: at D0, C57Bl/6 WT mice were instilled i.n with either PBS or IAV (300 pfu). Four days later, mice were further instilled i.t with either PBS or PAO1 (2.10^6^ cfu). At day 5, mice were sacrificed, and a bronchoalveolar lavage (BAL) was performed for cytospin cellular quantification **(H–K)**. Lungs were also obtained, inflated, cut and used for histological observation **(B–E)** and inflammation scoring **(F–G)**. NB: The three animals used for histology for the PBS/PAO1 and IAV/PAO1 groups all scored similarly, respectively 1 (*n* = 3) and 3 (*n* = 3). There are therefore no error bars. **(B–E)** (PBS/PBS; PBS/PAO1; IAV/PBS; IAV/PAO1, respectively): inflammatory lesions of treated lungs. Representative images of each condition on H&E slides at x20 original magnification. Scale bar: 200 μm. Stars indicate neutrophilic alveolar influx; arrow heads indicate perivascular and peribronchial mononuclear cell inflammation. Inserts are x40 magnification of cellular influx (mostly neutrophils in PBS-PAO1, mononuclear cells in IAV-PBS and a mix in IAV-PAO1). Results are shown as means ± SD. Statistical significance: ANOVA, multiple comparison, Tukey's test, with each point representing an individual mouse, *p < 0.05; **p < 0.01; ***p < 0.001; ****p < 0.0001.

In survival experiments, neither IAV (not shown) nor PAO1 alone (Figure 1A), induced any fatalities of C57Bl/6 mice, at the doses used. In contrast, IAV pre-treatment followed by PAO1 infection induced the death of all animals (Figure 1A).

In mechanistic experiments, C57Bl/6 mice infected with IAV (Figures 1D,F,G) or PAO1 (Figures 1C,F,G) exhibited increased lung tissue inflammation, compared to PBS mock-treated animals (Figures 1B,F,G). Neutrophils were the overwhelming cell type present after “PAO1 alone” infection, mainly in the alveoli (Figures 1C,F). By contrast, lymphocytes and monocytes (even though neutrophils were also present) were predominant in “IAV-alone”-infected animals and were mostly located in perivascular/peribronchial areas (Figures 1D,G).

**Figure 2:**
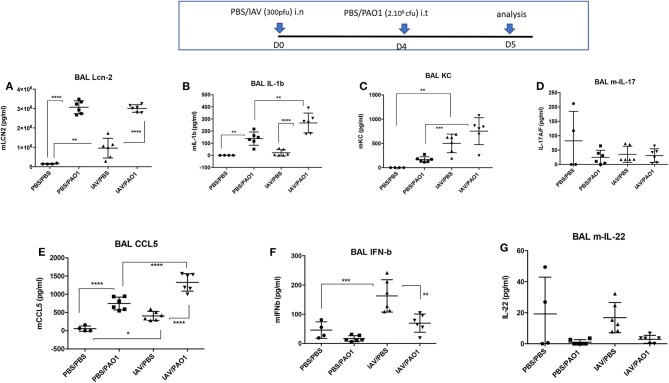
IAV lung pre-infection sensitizes WT C57Bl/6 mice to further PAO1-mediated cytokine production. BAL supernatants (see Figure 1 legend) were further used for assessment of cytokines and mediators. Each symbol represent an individual mouse. Statistical significance: ANOVA, multiple comparison, Tukey's test, with each point representing an individual mouse, *p < 0.05; **p < 0.01; ***p < 0.001; ****p < 0.0001. **(A)** Lcn-2 levels, **(B)** IL-1b levels, **(C)** KC levels, **(D)** IL-17 levels, **(E)** CCL5 levels, **(F)** IFN-b levels, **(G)** IL-22 levels.

Sequential IAV and PAO1 infections gave rise to increased neutrophilia, compared to “PAO1 alone” treatment (Figures 1E–G), especially in the peribronchial/perivascular areas, even though, notably, neither IL-17 nor IL-22 levels were increased over controls (Figures 2D–G).

This tissue inflammation was mirrored in BALs (Figures 1H–K), showing increased cytokine and inflammatory mediators in infected animals (IL-1β, KC, CCL-5, Lcn2, Figures 2A–C,E,F). IL-1β levels were only significantly increased following “PAO1-alone” and after “IAV+PAO1” infections (Figure 2B). With the notable exception of IFN-β, which was reduced, when compared to IAV alone (Figure 2F), and IL-17 and IL-22, which were not increased (Figures 2D,G), all inflammatory parameters were increased in IAV + PAO1-treated animals, compared to IAV alone. Compared to “PAO1” alone, IL-1β, KC and CCL-5 were significantly increased in the “IAV + PAO1” treatment.

**Figure 3:**
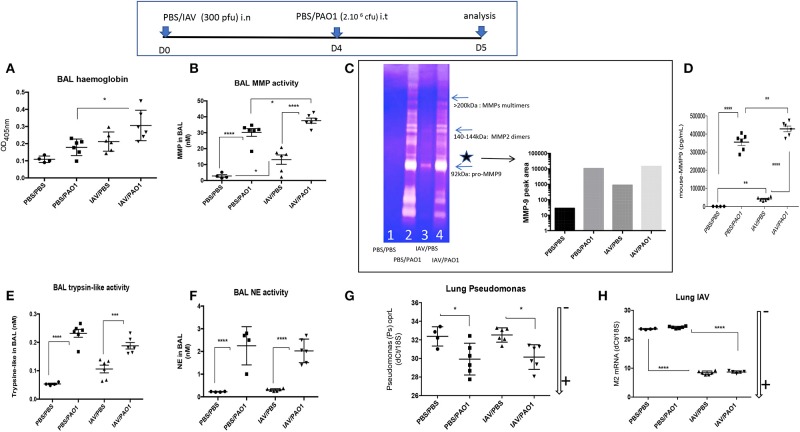
IAV lung pre-infection sensitizes WT C57Bl/6 mice to further PAO1-mediated metalloprotease activity and lung injury. BAL supernatants (see Figure 1 legend) was further used for hemoglobin content (absorbance at 405 nm, **A**) and protease activity **(B–H)**. **(B)** BAL metalloprotease bioactivity was assessed with the synthetic substrate 5-FAM-Ser-Pro-Leu-Ala-Gln-Ala-ValArg-Ser-Ser-Ser-Arg-Lys(5-TAMRA)-NH2 (35). **(C)** BAL metalloprotease bioactivity was also assessed by zymography. BALs from individual mice within the same experimental group (1: PBS/PBS; 2: PBS/PAO1; 3: IAV/PBS; 4: IAV/PAO1) were pooled and Pro-MMP-9 (*, left panel) intensity was assessed by densitometry (right panel). **(D)** BAL MMP-9 levels were measured by ELISA. **(E)** BAL trypsin-like activity was assessed with the synthetic substrate Boc-Phe-Ser-Arg-7-amido-4-méthylcoumarin (37). **(F)** BAL NE bioactivity was assessed with the synthetic substrate MeOSuc-Ala-Ala-Pro-Val-AMC (27). Lung extracts were also used for RNA preparation and RT-PCR analysis of M2 and oprL genes [as read-outs for IAV and PAO1 loads, respectively **(G–H)**]. Low and high RNA content is marked with an arrow indicating low (–) or high (+) level of expression. Results are shown as means ± SD. Statistical significance: ANOVA, multiple comparison, Tukey's test, with each point representing an individual mouse, *p < 0.05; **p <0.01; ***p < 0.001; ****p < 0.0001.

Inflammation resulted in lung injury, demonstrated by increased BAL hemoglobin levels, and again IAV pre-treatment potentiated the latter (Figure 3A). Potentially explaining this, BALF metalloprotease activity (MMP), as measured with a synthetic substrate, was increased in IAV-alone- and PAO1-alone-treated mice (Figure 3B). This activity was again potentiated, when IAV preceded PAO1 treatment. Using zymography, we further showed that MMP-9 was present and was clearly the most abundant MMP in BAL of IAV-infected animals (^*^, 3C), which was confirmed by ELISA (3D). Importantly, and specifically, IAV/PAO1 induced more MMP-9 than PAO1 alone (3D).

Other protease activities were also present in BAL, albeit at much lower concentrations: trypsin-like activity was mostly increased in PAO1-alone and IAV+PAO1 arms of the experiment (Figure 3E), compared to MMP levels.

NE BALF activity was also increased in PAO1-alone and in IAV+PAO1-treated mice, but very poorly in “IAV-alone” mice (Figure 3F).

Notably, neither IAV nor PAO1 influenced each other's infection, using M2 and oprL genes as a read-out for IAV and PAO1 loads, respectively (Figures 3G,H), suggesting that dysregulated direct antimicrobial activity was not a major player here.

### Epithelial Cell Modeling of IAV/PAO1/IL-1b Stimulations

Because epithelial cells are the main IAV targets, the interactions studied above *in vivo* were then modeled *in vitro* in these cells. After a comparative study in NCI-H292, BEAS-2B, and A549 lung cells, we found (not shown) that A549 cells were most responsive to IAV-, IAV PAMPs-, PAO1-, and IL-1ß-mediated infection/stimulation, and these cells were therefore further studied below:

(a) IAV differentially regulate inflammatory/anti-viral and antimicrobial mediators in A549 cells.

We either infected A549 cells with IAV (moi 1) or stimulated them with either poly IC, 5′ ppp ds RNA, synthetic ligands for TLR-3, RIG-I and MDA-5, respectively, or with IL-1ß, a sterile inflammatory stimulus. We then measured the RNA and protein levels of a variety of mediators (cytokines, antimicrobial/anti-inflammatory molecules), all relevant to the *in vivo* model described in Figures 1–3.

**Figure 4:**
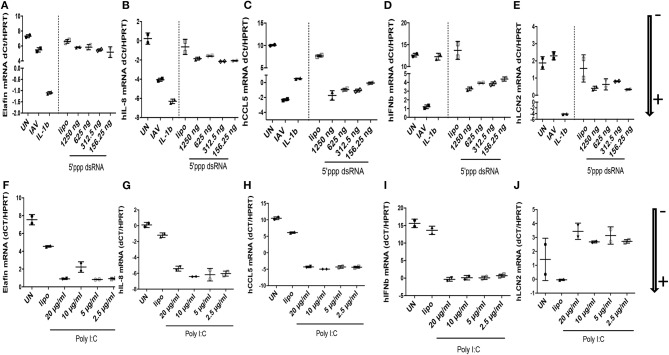
IAV-, polyIC-, 5′ppp dsRNA-, and IL-1β- mediated induction of elafin, IL-8, CCL5, IFN- β, Lcn-2 RNA in A549 cells. At D0, A549 cells (24 wells plates, 70% confluence) were either left unstimulated (UN), infected with IAV (moi 1), or stimulated with either IL-1β (10 ng/ml), lipofectamine control (CLT lipo), 5′ ppp dsRNA (Invitrogen) or poly IC (Invitrogen), at various concentrations during 4 h at 37°C in OPTI-MEM medium. Cells were then washed, medium replenished, and 16 h later, cell lysates were recovered for further analysis of RNA **(A–J)**, respectively, for elafin, IL-8, CCL5, IFN- β, Lcn-2. q-PCR analysis was performed, and results expressed as dCT (CT gene of interest-CT HPRT), with low and high values representing high and low levels of the gene of interest, respectively (marked with an arrow indicating low (–) or high (+) level of expression). Two independent experiments (individual symbols) were performed. Results are shown as means ± SD.

At the RNA level (Figure 4: NB: ΔdCT values are inversely proportional to RNA levels, and arrows indicate gene expression +/–), we showed that IL-8 was clearly induced by IAV and IL-1ß (ΔdCT of −4 and −7.5 respectively, Figure 4B), but modestly by 5′ ppp dsRNA (ΔdCT of −1.0, Figure 4B) and at intermediate levels by poly-IC (ΔdCT of −4, Figure 4G). By contrast, CCL-5 RNA was robustly induced by all stimuli (ΔdCT of respectively −12, −10, −9.5, and −10, Figures 4C,H). Lcn-2 RNA was also strongly up-regulated by IL-1ß (ΔdCT of −7, Figure 4E), modestly by 5′ ppp dsRNA (Figure 4E) and even inhibited by poly IC (Figure 4J).

IFN-ß RNA stood alone since it was not induced by IL-1ß (Figure 4D), but was, as expected, strongly up-regulated by IAV, 5′ ppp dsRNA, and polyIC (ΔdCT of −11.5, −8, and −15, respectively, Figures 4D,I). When elafin, an antimicrobial molecule with anti-inflammatory/anti-NF-kb activity (27–30) was considered, IL-1ß was again clearly the greatest inducer of elafin mRNA (ΔdCT of −8, Figure 4A), with polyIC and IAV being strong and intermediate inducers (ΔdCT of −6.5 and −2, Figures 4A,F), respectively.

**Figure 5:**
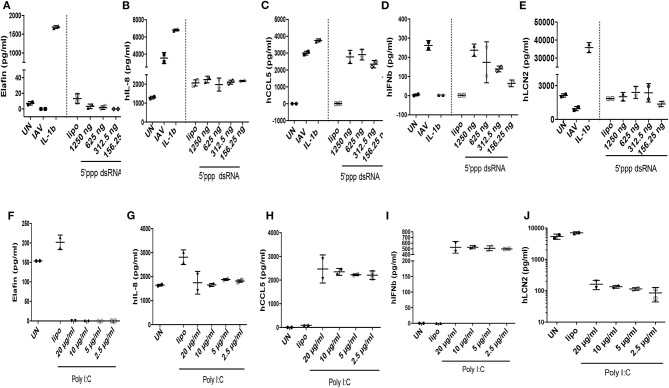
IAV-, polyIC-, 5'ppp dsRNA- and IL-1β- mediated induction of elafin, IL-8, CCL5, IFN- β, Lcn-2 protein in A549 cells. The supernatants obtained from the experiments depicted in Figure 4 were recovered and used for protein measurement (ELISA) of elafin, IL-8, CCL5, IFN- β, Lcn-2 **(A–J)**.

When protein levels were studied (Figure 5), we observed that IAV had important post-transcriptional regulatory activities: IAV clearly up-regulated IL-8, CCL-5, IFN-ß proteins (Figures 5B–D, respectively), in keeping with increased RNA levels, while having no effect on elafin and Lcn2 proteins (Figures 5A,E, respectively). Poly IC and 5′ ppp dsRNA effects were even more contrasted: poly IC slightly down-regulated the accumulation of IL-8 (Figure 5G) and drastically down-regulated that of elafin and Lcn-2 (Figures 5F,J) proteins, while very robustly inducing that of CCL-5 (Figure 5H) and IFN-ß (Figure 5I). Similarly, 5′ ppp dsRNA also sharply induced CCL-5 and IFN-ß protein accumulation (Figures 5C,D, respectively), in keeping with its effect on RNA, but had virtually no effect on elafin (Figure 5A), IL-8 (Figure 5B), or Lcn2 (Figure 5E) protein accumulation. These results demonstrated that IAV had specific down-regulatory post transcriptional activity on elafin and Lcn-2, and that its effect is mimicked by its nucleic acid analogs, either polyIC or 5′ppp dsRNA, suggesting an intra-cellular mode of action:

(b) IAV, but not PAO1, post-transcriptionally down-regulates elafin expression in A549 cells.

We then demonstrated in further *in vitro* independent experiments that the post-transcriptional regulation observed in Figures 4, 5, in which IAV induced RNA, but not protein levels of elafin, was indeed “IAV-specific,” since PAO1 up-regulated both RNA and protein levels of elafin and IL-8 instead (see Figures S1G,H,J,K and the Supplementary Materials).

(c) IAV exacerbates IL-1ß- and PAO1-mediated inflammatory responses and down-regulates elafin and Lcn-2 accumulation in A549 cells.

**Figure 6:**
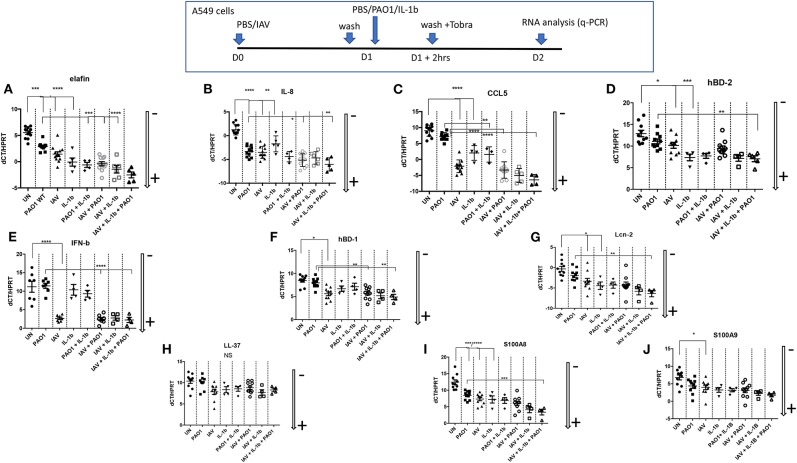
Epithelial cell regulation of cytokines/chemokines and antimicrobials transcription in IAV/PAO1/IL-1β co-infection models. At D0, A549 cells (24 wells plates) were either uninfected or infected with IAV (moi 1) in serum-free medium for 1 h. After washing, and o/n incubation, cells were either replenished with fresh medium and left untreated (UN), or treated with either PAO1 alone (moi 1), IL-1β alone (10 ng/ml), or IL-1β + PAO1 (at the same doses). After a further 2 h, cells were washed with F12K medium containing tobramycin (40 μg/ml), to remove PAO1 load, and left for 24 h in medium. At the end of the incubation, at D2, supernatants and cell lysates were recovered, and cytokines/chemokines/antimicrobials were assessed for RNA (this figure) and protein levels (ELISAs, Figure 7). For RNA analysis, gene expression is marked with an arrow indicating low (–) or high (+) level of expression (see Figure 4 for the full explanation). Results are shown as means ± SD. Statistical significance: ANOVA, multiple comparison, Tukey's test, with each point representing an individual mouse, *p < 0.05; **p < 0.01; ***p < 0.001; ****p < 0.0001. **(A)** Elafin RNA, **(B)** IL-8 RNA, **(C)** CCL5 RNA, (D) hBD-2 RNA, **(E)** IFN-b RNA, **(F)** hBD-1 RNA, **(G)** Lcn-2 RNA, **(H)** LL-37 RNA, **(I)** S100 A8 RNA, **(J)** S100A9 RNA.

**Figure 7:**
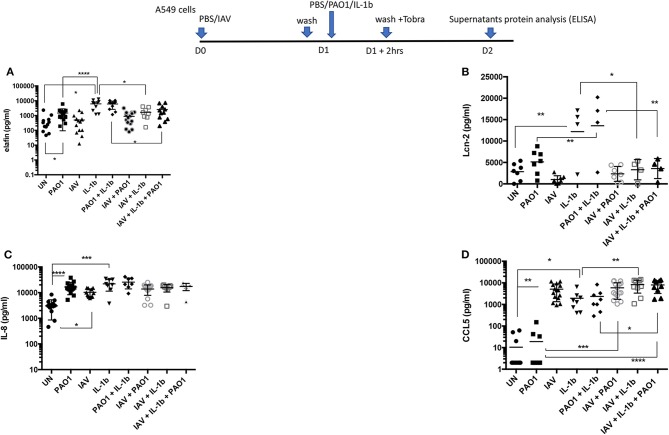
Cytokines/chemokines and antimicrobials protein production in IAV/PAO1 and IAV/ IL-1β co-infection models. Elafin, IL-8, CCL5, and Lcn-2 protein content was assessed by ELISA in the supernatants from the experiment described in Figure 6. Results are shown as means ± SD. Statistical significance: ANOVA, multiple comparison, Tukey's test, with each point representing an individual mouse, *p < 0.05; **p < 0.01; ***p < 0.001; ****p < 0.0001. **(A)** Elafin levels, **(B)** Lcn-2 levels, **(C)** IL-8 levels, **(D)** CCL5 levels.

Having shown *in vivo* that IAV could exacerbate PAO1 responses (Figures 1–3) and that, studied individually, IAV alone (but not PAO1) could inhibit *in vitro* elafin and Lcn-2 protein production specifically (Figure 5 and Figure S1), we set up a “multi-hit inflammatory model” in A549 cells, where combinations of “mixes” were studied together (Figures 6, 7), similarly to the *in vivo* protocol. In addition to IAV and PAO1, IL-1ß was added in this model, as an important “first wave” cytokine up-regulated *in vivo* (Figure 2).

Echoing these *in vivo* data, we showed that all these agents up-regulated the transcription of many inflammatory/antimicrobial molecules in A549 cells, as evidenced by a reduction in dCT levels (Figure 6). Notably, compared to “PAO1 alone,” pre-infection with IAV followed by PAO1 infection further increased IL-8 (Figure 6B), CCL-5 (Figure 6C), hBD-1 (Figure 6F), and elafin (Figure 6A) mRNA levels.

IFN-ß inductions stood out as notable exceptions (Figure 6E), where only IAV-containing “mixes” were effective agonists, and LL-37 (Figure 6H), whose expression was relatively stable, in keeping with the described relative constitutiveness of this antimicrobial.

When protein levels were assessed in supernatants (Figure 7), IAV again demonstrated post-transcriptional regulation on elafin and Lcn-2; indeed, there was a trend for decreased elafin and Lcn-2 production in IAV + PAO1 treatments, compared to PAO1 alone, and IAV had a clear down-regulating effect on these mediators when “IAV+ IL-1β” and “IAV+ PAO1 + IL-1β” were compared to “IL-1β” and “IAV+ PAO1,” respectively (Figures 7A,B). Again, this regulatory effect was specific since, as demonstrated above, IAV clearly up-regulated IL-8 and CCL-5 proteins, compared to untreated cells, and did either not change for IL-8 (Figure 7C) or even increased for CCL-5 (Figure 7D) protein production, when cells were infected with IAV and further treated with PAO1 and/or IL-1β.

**Figure 8:**
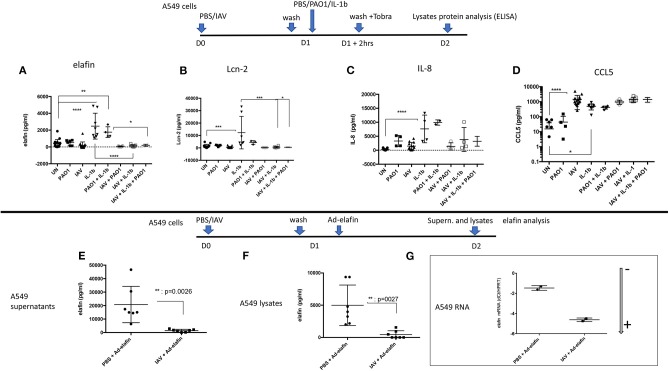
IAV-mediated post-transcriptional regulation occurs intra-cellularly and in an Adenovirus-elafin reporter system in A549 cells. Top panel: A549 cells were treated as explained in the Figure 6 legend. Cell lysates were recovered and protein levels for elafin, Lcn-2, IL-8, CCL-5 (**A–D**, respectively) were measured by ELISA. Bottom panel: A549 cells were mock-infected or infected with IAV (moi =1), as explained above. After 16 h, cells were further infected with replication-deficient Adenovirus-h-elafin (33). After a further 24 h, A549 supernatants and lysates were recovered and elafin protein levels measured by ELISA (**E,F** respectively). In a subset experiment, cell lysates were recovered for q-PCR assessment of elafin RNA content. The latter is marked with an arrow indicating low (–) or high (+) level of expression **(G)** (see Figure 4 for full explanation). Results are shown as means ± SD. Statistical significance (except for **G**): ANOVA, multiple comparison, Tukey's test, with each point representing an individual mouse, *p < 0.05; **p < 0.01; ***p < 0.001; ****p < 0.0001).

To assess whether the IAV regulatory effect was acting intra- or extra-cellularly, the levels of the same mediators were measured in A549 lysates (instead of in supernatants). We found that, mirroring the effects observed in A549 supernatants, IAV again down-regulated the IL-1β-mediated intracellular accumulation of elafin and Lcn2 (Figures 8A,B), but not those of IL-8 and CCL-5 (Figures 8C,D).

In addition, we also studied the IAV regulation of an exogenously added Adenovirus-h-elafin construct in A549 cells (Figures 8E–G) and found that IAV also down-regulated elafin accumulation in both supernatants and lysates (Figures 8E,F).

Importantly, this IAV-mediated down-regulation was not due to an interference of IAV with Ad infection, since IAV + Ad-h-elafin- RNA levels were even higher than in the Ad-elafin “alone” condition, as demonstrated with lower dCT levels; showing that the Ad vector efficiently delivered its genetic cargo (Figure 8G).

### IAV Pre-Infection Down-Regulate Elafin Expression *in vivo* and Exacerbates *P. aeruginosa* Inflammation in Elafin-Over-Expressing Mice

**Figure 9:**
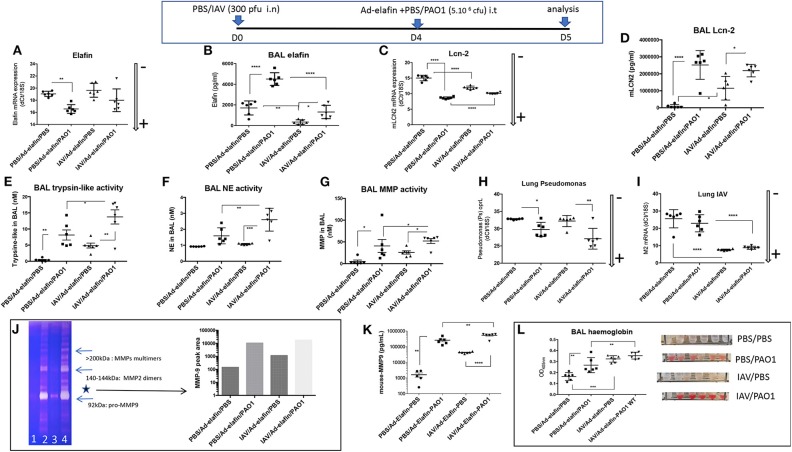
IAV lung pre-infection sensitizes elafin over-expressing mice to further PAO1-mediated inflammation, metalloprotease activity, and lung injury. At D0, C57Bl/6 WT mice were instilled i.n with either PBS or IAV (300 pfu). Four days later, mice were further instilled i.t with Ad-elafin (3.10^6^ pfu) plus either PBS or PAO1 (5.10^6^ cfu). At day 5, mice were sacrificed, and a bronchoalveolar lavage (BAL) was performed. Lungs were also obtained, and RNA prepared for q-PCR assessment of elafin and Lcn-2 **(A,C)**. BAL was also used for elafin and Lcn-2 protein measurements **(B,D)**, and protease activity **(E–G)**, using synthetic substrates, or zymography **(J)**, as explained in the Figure 3 legend. **(E)** BAL trypsin-like activity; **(F)** BAL NE bioactivity; **(G)** BAL MMP bioactivity; **(H)** Lung PAO1 load was assessed by q-PCR by measuring oprL expression; **(I)** Lung IAV load was similarly measured by q-PCR (M2 gene). As for other Figures, gene expression is marked with an arrow indicating low (–) or high (+) level of expression. **(J)** MMP bio activity was assessed by zymography: BALs from individual mice within the same experimental group (1: PBS/PBS; 2: PBS/PAO1; 3: IAV/PBS; 4: IAV/PAO1) were pooled and Pro-MMP-9 (*, left panel) intensity was assessed by densitometry (right panel). **(K)** BAL MMP-9 levels were measured by ELISA. **(L)** Hemoglobin levels were measured by absorbance at 413 nm. Results are shown as means ± SD. Statistical significance: ANOVA, multiple comparison, Tukey's test, with each point representing an individual mouse, *p < 0.05; **p < 0.01; ***p < 0.001; ****p < 0.0001.

Having demonstrated *in vitro* that IAV specifically inhibited elafin and Lcn-2 protein production in lung epithelial cells, two important antimicrobial/anti-inflammatory molecules (27–30, 38), we then tested whether this regulatory effect was also observed *in vivo*. Because C57Bl/6 WT mice are “natural KO” for elafin (34), this was tested in h-elafin over-expressers, using the Ad-h-elafin over-expressing system (33). Since elafin expression was only required in our protocol as a “read out target” for IAV, Ad-h-elafin was only given 16 h (at the same time as either PBS or PAO1) before animals were culled for analysis. Expectedly, given its anti-inflammatory nature as demonstrated previously (27–30), the PBS/Ad-elafin/PBS “Control” arm of the experiment did not induce any inflammation “per se,” as assessed by a “typical” percentage of macrophages and neutrophils recovered in BALs, 99 and 1%, respectively, Figures S2B–D. Importantly, although IAV did not decrease either basal or PAO1-induced lung elafin RNA levels (Figure 9A), it drastically reduced elafin protein accumulation in BALs, akin to that observed *in vitro* (Figure 9B). In contrast, IAV up-regulated both Lcn-2 RNA and protein levels (Figures 9C,D) and did not significantly affect PAO1-mediated Lcn-2 protein up-regulation (Figure 9D), contrary to that observed *in vitro* in epithelial cells.

**Figure 10:**
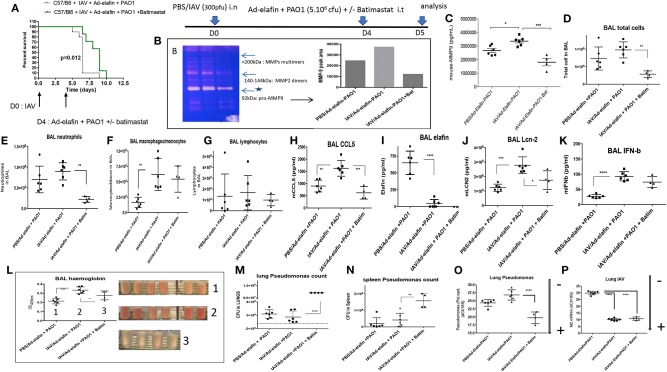
The metalloprotease batimastat delays lethality in IAV + PAO1 infection and reduces inflammation and lung injury. **(A)** Survival experiment: C57/Bl6 mice were instilled i.n with IAV (300 pfu). Four days later, mice were further instilled i.t with Ad-elafin (3.10^6^ pfu) and PAO1 (1.10^5^ cfu) +/– the metalloprotease inhibitor batimastat (200 μgs). Survival was then followed and analyzed using Kaplan-Meier curves. Statistical tests were performed with the GraphPad Prism 6 package, using the Log-rank (Mantel-Cox) test. **(B–N)** Mechanistic experiments: C57Bl/6 WT mice were instilled i.n with either PBS or IAV (300 pfu). Four days later, mice were further instilled i.t with Ad-elafin (3.10^6^ pfu) and PAO1 (5.10^6^ cfu) +/– batimastat (200 μgs). At day 5, mice were sacrificed, and a bronchoalveolar lavage (BAL) was performed. BAL fluid supernatant was used for MMP measurements (zymography, **B**, and MMP-9 ELISA, **C**), cystospin cellular quantification **(D–G)**, ELISA assessment of a variety of mediators **(H–K)**, hemoglobin measurements **(L)**. Lungs and spleens were also harvested and used to measure PAO1 load (cfu counts on agarose plates, **M** and **N**, respectively). NB: in **(M)**, bacteria numbers were too high to be counted with the required precision. The value was therefore set at the maximum countable, considering the dilution used, i.e., 10^8^ bacteria. PAO1 and IAV load were also independently assessed by measuring by q-PCR oprL and M2 gene expression, **O** and **P** respectively. Gene expression is marked, as above, with an arrow indicating low (–) or high (+) level of expression. Results are shown as means ± SD. Statistical significance: ANOVA, multiple comparison, Tukey's test, with each point representing an individual mouse, *p < 0.05; **p < 0.01; ***p < 0.001; ****p < 0.0001.

Irrespectively, infection of Ad-h-elafin-treated mice with either IAV or PAO1 exhibited increased inflammation, as assessed by BAL total inflammatory cells, neutrophilia (Figures S2A–F), increase in cytokine and antimicrobials levels (Figures S2G–N), protease activity (Figures 9E–G,J), and tissue injury (Figure 9L). This confirmed what was observed in WT C57Bl/6 mice (Figures 1–3), i.e., the exacerbated effect of IAV on PAO1 infection and the key involvement of metalloproteases, including MMP-9 (Figures 9G,J). Importantly again, as also demonstrated in WT C57Bl/6 mice (Figure 3G), neither IAV nor PAO1 influenced each other's infection (Figures 9H,I), reinforcing that dysregulated direct antimicrobial activity was not the major cause of IAV-induced inflammatory exacerbations. This was further strengthened by the use of the MMP inhibitor batimastat (see below, Figure 10). The latter was chosen because it does not inhibit LasB (39), quantitatively the most abundant PAO1 metalloprotease (30, 35), therefore allowing us to specifically address the effect of IAV on host (and not PAO1) metalloproteases. Importantly, because host proteases are known to be important for IAV replication (40, 41), and in order not to affect that cycle, batimastat was given “therapeutically” at D4 at the peak of IAV replication, at the same time as PAO1, and not “prophylactically” at the time of IAV infection (D0).

We showed that batimastat delayed the lethality of mice co-infected with IAV and PAO1 (Figure 10A), and down-regulated gelatinolytic activity, including MMP-9 expression, as shown by zymography (Figure 10B), and ELISA (Figure 10C). In addition, batimastat treatment down-regulated neutrophilic inflammation (Figures 10D–G) and tissue injury (Figure 10L), confirming that MMPs are indeed instrumental in the IAV-subversion of lung tissue resilience.

Interestingly, the beneficial effect of batimastat did not extend to rescuing elafin protein levels (Figure 10I), and even down-regulated Lcn-2 accumulation (Figure 10J), suggesting that proteolytic digestion of elafin by MMPs was not at play here, neither *in vivo* nor *in vitro* (not shown). Interestingly, batimastat was strikingly associated with a sharp increase in PAO1 load in lungs (Figures 10M,O), assessed by two independent methods, and in spleen (Figure 10N), demonstrating bacterial translocation into the periphery, and suggesting again a clear dissociation increase between tissue resilience and resistance to PAO1. In contrast, batimastat did not influence IAV load (Figure 10P). Some of the above results are described in more detail in the Supplementary Material (Supplementary Material Results and Figure S2).

## Discussion

Bacterial superinfections are an established risk of primary viral infections (e.g., rhinovirus, *Influenza* virus). Among the many potential mechanisms advocated, previous studies have suggested that IAV might promote further bacterial outgrowth by down-regulating neutrophilic function (14–17) or antimicrobial molecules (18–20). Notably, most authors have modeled their studies using *S. aureus* or *S. pneumoniae* as the secondary bacterial “hit,” and despite its obvious clinical importance, no studies have, to our knowledge, comprehensively investigated mechanisms linking *Influenza* (IAV) and *P. aeruginosa* infections (16, 19). Using the latter combination, with the H3N2 and PAO1 strains, respectively, we show here in a variety of *in vitro* and *in vivo* models, that IAV dramatically down-regulates, at the post-transcriptional level, the antimicrobial/anti-inflammatory elafin/trappin-2 *in vitro* (Figures 4–8 and Figure S1) and *in vivo* in PAO1- (Figures 9, 10 and Figure S2) and IL-1β-mediated models of inflammation (Figures S3, S4 and Supplementary Material Results). Unfortunately, our attempts to determine whether other antimicrobial molecules might also be inhibited by IAV were thwarted by the previously reported unreliability of current available ELISA kits for antimicrobial molecules (not shown). An important exception was Lcn-2, which we also showed to be inhibited by IAV *in vitro* in epithelial cells (Figures 4, 5, 7), but not, unlike elafin, *in vivo* in mice lungs (Figures 9, 10 and Figure S3). Although the exact mechanism still remains obscure, IAV likely down-regulates, at least *in vitro*, an epithelial intra-cellular/cytosolic event, since elafin and Lcn2 intra-cellular protein levels were also drastically reduced (Figure 8).

Relatedly, in a previous study, Robinson et al. also showed that pre-infecting C57Bl/6 mice with PR8 H1N1 IAV down-regulated the antimicrobials Lcn2, RegIIIγ, and S100A8 mRNA levels upon further *S. aureus* infection, but the effect on protein levels was not reported, again likely because of the paucity of reliable ELISA kits (18). Relevantly, Mallia et al. showed that rhinovirus induced neutrophil elastase in COPD patients and suggests that the ensuing down-regulation of secretory leukocyte protease inhibitor and elafin is causative in triggering exacerbations in these patients (20). Relatedly, although no bacterial data were reported, in a transcriptomic study enrolling 1,610 individuals, 142 of which were followed for evaluation of acute viral respiratory illness, the elafin gene (PI3) was found to be the top downregulated gene in the acute phase of the *Influenza* infection, but not in the rhinovirus or other infection groups (42).

Regardless of the mechanisms, a reduction in some antimicrobial molecule levels, like elafin in our study, is certainly a plausible mechanism to explain further sensitivity to bacterial infections. However, although it has long been assumed that these molecules only have a direct bacteriostatic/bactericidal activity on microbes, it is now apparent that they have more complex and pleiotropic activities (43). Specifically, we have previously demonstrated that a 5 day local over-expression of elafin protected mice lungs against maladaptive neutrophilic inflammation in *P.a* infections (27, 28, 30), and also against Plasmodium-induced inflammation, through the induction of anti-inflammatory pathways (29). Importantly, the focus of the present study was not to “re-demonstrate” the protective effect of elafin against *P.a* (see above, 27), since elafin expression was short-lived in our Ad-elafin *in vivo* protocol (16 h), but allowed us, as discussed above, to demonstrate for the first time that it is down-regulated by IAV.

Equally as important, and indeed the initial focus of our study, was the demonstration that IAV pre-infection exacerbated further PAO1-mediated inflammatory responses, regardless of elafin presence (Ad-elafin protocol, Figure 9 and Figure S2), or of its absence (in C57Bl/6 WT mice, Figures 1–3). Indeed, in the context of IAV+ PAO1 infection, increased lung inflammatory cell influx, particularly neutrophils, which were activated, as evidenced by increased NE bioactivity, was associated with enhanced inflammatory markers, e.g., IL-1β, KC, and with an increase in protease (mainly MMP) activity, and with tissue injury.

Strengthening previously reported data that IAV can induce metalloproteases in the lung and other organs (44–48), we further demonstrated that induced MMP activity (including that of MMP-9) by IAV pre-infection was indeed likely an important factor in sensitization of mice to further PAO1-mediated lung damage, since the MMP inhibitor batimastat significantly delayed lethality (Figure 10A) and diminished inflammatory responses (Figures 10D–G) and tissue damage (Figure 10L).

Although the cellular source of MMPs was not investigated here, neutrophils are known to secrete MMP-2 and 9 and they were likely a significant source (48). Interestingly, there was very little NE bio-activity in BALs from “IAV-alone”-infected animals, suggesting that either MMPs are more readily secreted than NE post-IAV, or that “IAV-alone” induced the secretion of elastase inhibitors capable of blocking NE activity.

Importantly, in contrast to previous IAV/bacteria associations studied in the past, e.g., IAV/*S.aureus*; IAV/ *S. pneumoniae*, IAV pre-treatment did not condition the host to further PAO1 infection by restraining the IL-1β-IL-17 pathway (21, 22), and/or neutrophilic responses to bacteria (14–17). Although these differences may partly be explained by differences in the strains of *Influenza* used (H3N2 Scotland here), we believe that *P.a* may clearly respond differentially from other bacteria. This combination sets the scene for a furthered deleterious protease (MMP mainly) response and a down-regulation of elafin, a key anti-inflammatory molecule, resulting in increased tissue injury, where neutrophils probably play a major role (37, 49, 50).

In conclusion, as demonstrated by the fact that bacterial growth and dissemination does not equate with decreased survival (Figure 10), our data strengthen the concept (26, 51) that improvement of tissue resilience by inhibiting host proteases [(27–30), this study] and up-regulating antimicrobials/anti-inflammatory molecules inhibited by IAV, such as elafin (27–30) is not necessarily associated with bacterial clearance (specifically *P.a* in our study). Indeed, the MMP inhibitor batimastat even promoted bacterial dissemination, suggesting that MMPs might have anti-bacterial activities. This may have to be carefully considered in clinical situations where IAV/*P.a* co-infections are found (VAP, cystic fibrosis…).

## Data Availability Statement

The datasets generated for this study are available on request to the corresponding author.

## Ethics Statement

Procedures involving mice were approved by our Ethical Committee (Paris-Nord/No 121) and by the French Ministry of Research (Agreement Nos. 4537.03 and 02012).

## Author Contributions

BV, BS, MS, and FL performed experiments. AC performed histological analysis. IG-V helped in the design of the experiments and critically appraised drafts of the document. J-MS designed experiments, analyzed data, and wrote the manuscript.

### Conflict of Interest

The authors declare that the research was conducted in the absence of any commercial or financial relationships that could be construed as a potential conflict of interest.
