# Supplementary material for: Influenza A Virus Pre-Infection Exacerbates Pseudomonas aeruginosa-Mediated Lung Damage Through Increased MMP-9 Expression, Decreased Elafin Production and Tissue Resilience
Source: Front Immunol. 2020 Feb 13;11:117. doi: 10.3389/fimmu.2020.00117 (PMC7031978; doi:10.3389/fimmu.2020.00117)
Supplement: Supplementary file 1 [file Data_Sheet_1.pdf]

Supplementary Material

Results

1) IAV, but not PAO1, post-transcriptionally down-regulates elafin expression in A549 cells.

Having demonstrated a post-transcriptional regulation of elafin *in vivo* (Fig 9), PAO1 and IAV infections were then compared side by side at various time points in A549 cells.

We confirmed, as found above (Figs 4-5), that IAV induced elafin mRNA *in vitro* (but only at the 16hrs time point, FigS1, panel A), and that there was no induction of protein (Fig S1, panel E). We also confirmed, as shown before (Figs 4-5) that IAV induced both IL-8 mRNA and proteins at the 16hrs time point (Fig S1 panels B, F). As above, IL-1β was also a strong inducer of both elafin and IL-8 mRNA and protein levels (Fig S1 panels A, E and B, F, respectively). IAV was also a very strong inducer of IFN-β mRNA (Fig S1 panel C).

When PAO1 induction of A549 was considered (note that the 16hrs time point was discarded from the analysis because of high PAO1 cytotoxicity at that time point), the pattern of expression of the mediators was clearly different : PAO1 was able to induce the expression of RNA and protein for both elafin (Fig S1, panels G and J) and IL-8 (panels H and K), but no expression of IFN-β mRNAs was noted (Fig S1, panel I).

Importantly, both live IAV and PAO1 were necessary for these inductions since when inactivated IAV (IAV*) or PAO1 (PAO1*) were used instead (the efficiency of this inactivation was checked for the former by measuring IAV replication, which was completely inhibited, as evidenced by absence of M2 read-out, panel D), no gene/protein regulation was observed. Altogether, these data demonstrate that, as as found *in vivo*, IAV, but not PAO1, down-regulates elafin at the post-transcriptional level.

2) *In vivo* IAV pre-infection exacerbates *P.aeruginosa* inflammation in elafin-over-expressing mice.

In mechanistic experiments, we showed that as in WT C57Bl/6 mice (Fig 1), neutrophils were the overwhelming cell type present after PAO1 infection of Ad-elafin-treated mice (Fig S2, panels C, E, F), whereas lymphocytes (even though neutrophils were also present) were increased in IAV-infected animals (Fig S2, panel D). This was paralleled by increased BAL cytokine and inflammatory mediators production in infected animals (IL-1β, KC, CCL-5, panels G-I). Notably, the levels of cytokines induced differed notably between treatments: IL-1β and KC levels were only significantly increased following PAO1 infection (Fig S2, panels G-H), whereas IFN- β was only increased following IAV infection (panel J). CCL5 was induced by both IAV and PAO1 (Fig S2, panel I). Notably, when further antimicrobial molecules were considered (mS100A8, mS100A9, mREG3g, mCAMP), PAO1 infection drastically increased their transcription (as noted by a sharp decrease in dCTs, Fig S2, panels K-M), with the exception of REG3g (panel N). By contrast, their induction by IAV was less marked for mLCN2, mS100A8, mS100A9.

When sequential IAV and PAO1 infections were analysed, inflammation was exacerbated, when compared to individual IAV or PAO1 treatments (Fig S2). Indeed, with the notable exception of IFN- β (which was reduced, when compared to IAV alone), most of inflammatory mediators assessed above were increased in the IAV + PAO1 arm of the experiment, when compared to 'IAV' alone or 'PAO1 alone' groups (Fig S2).

3) IAV pre-infection down-regulates elafin expression in elafin-over-expressing mice in an IL-1β-mediated sterile inflammation model

Having shown that IAV could exacerbate PAO1 responses in two *in vivo* independent models (C57Bl/6 WT mice and Ad-elafin-treated C57Bl/6 WT mice), its effect was then tested in a

sterile IL-1β-mediated model of inflammation, also using Ad-elafin as a reporter elafin-expressing system (see Figs S3-S4).

C57Bl/6 WT mice were pre-infected at D0 with IAV, and 4 days later, were treated intra-tracheally with Ad-elafin and either PBS or IL-1β (100 ng). After a further 16hrs, mice were sacrificed, BAL performed, and lung tissue processed. The levels of a variety of cytokines and mediators (including elafin) were measured by q-PCR and ELISA and BAL cellular inflammation was assessed by performing cytospins.

As above (PAO1 experiment, Fig S2 A-D), during this very short timing (16hrs), elafin expression on its own did not induce any inflammatory responses (Fig S3, panels E-H).

Notably, as above in the 'PAO1 model' (Fig S2), and in epithelial cells *in vitro* (Fig 5), IAV increased the transcription of most antimicrobials (Fig S2, panels J-L), except that of elafin (panel A) and Reg3g (panel I), whereas IL-1β enhanced all mRNA levels, except for that of Reg3g (Fig S3, panel I). As noted before in the 'PAO1 models' *in vivo* and in epithelial cells *in vitro,* IAV clearly down-regulated BAL protein levels of elafin, but not that of Lcn-2 (Fig S3, panels B, D).

Although IL-1β treatment did induce some inflammation, as evidenced by increased cell influx in BAL (and neutrophils in particular, Fig S3, panels E-H, which correlated with KC levels, Fig S4 panel B), its intensity was clearly lower than when using PAO1 as a 'stimulus' (Fig S2). In particular, IL-1β on its own did not induce proteolytic activity (not shown), and concomittantly, very little tissue lung injury (as measured by measurement of BAL levels of haemoglobin) was noted (Fig S4, panel F : note the very low OD values, compared to 'PAO1 experiments').

When sequential IAV and IL-1β treatment was considered, BAL cell influx (mostly neutrophils and lymphocytes) was increased, compared to IL-1β alone (Fig S3, panels E-H). Interestingly however, on a background of IAV infection, as with the 'PAO1 models' (Fig 2

and S2), IL-1β treatment decreased lymphocyte numbers, compared to ‘IAV only’ (Fig S4, panel E).

Also, as demonstrated above in the other models, inflammation (represented here by the IL-1β treatment) did not influence IAV persistence in the lungs of mice, using M2 PCR as a read-out for IAV replication (Fig S4, panel G).

Supplementary Figures

Fig S1A

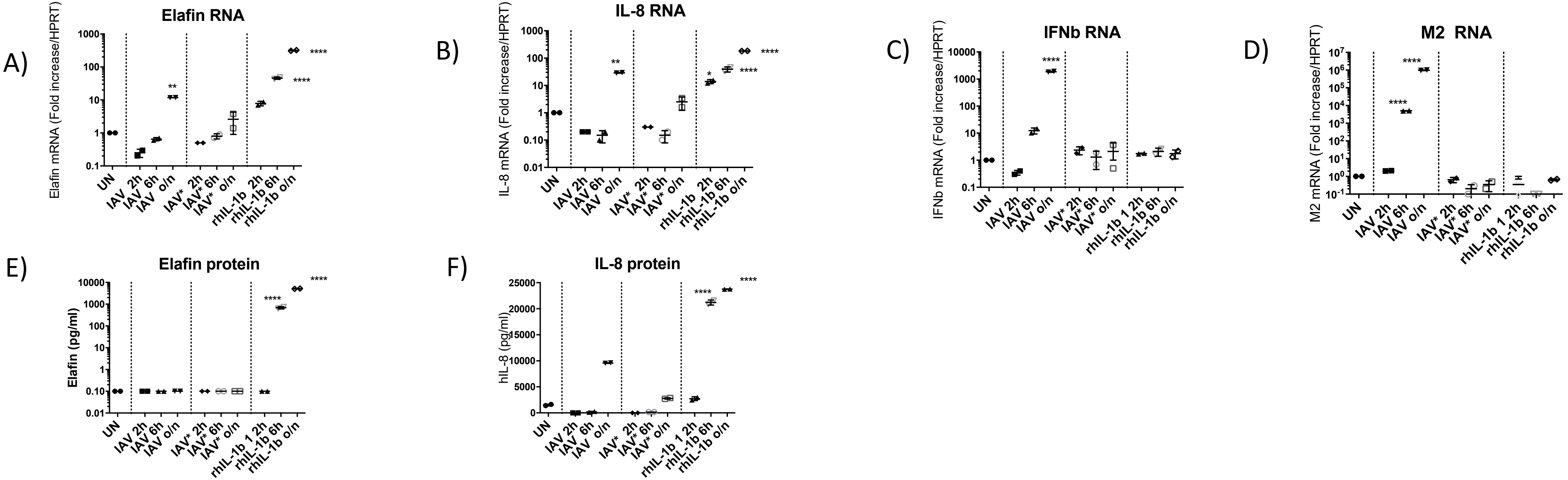

Fig S1B

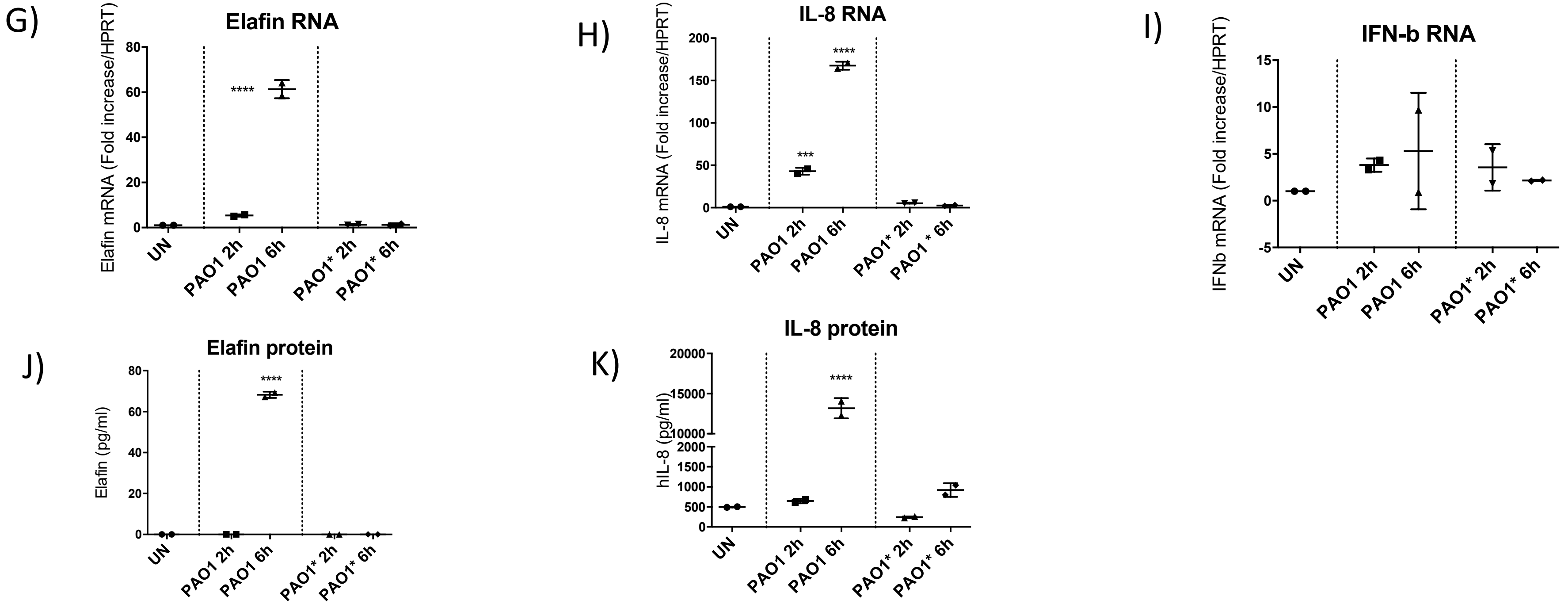

Fig S1 Pseudomonas aeruginosa up-regulates elafin mRNA and protein levels in A549 cells

Top panel : A549 cells were either treated with IL-1β (10ng/ml) or infected with live- or heat-inactivated (*)-IAV (moi =1) in MEM medium during either 2hrs, 6hrs, or o/n. Cell lysates were then recovered for q-PCR quantification of RNA (elafin, IL-8, IFN-β, M2), using the following formula : fold increase: (RQ) = $2^{-(\Delta\Delta CT)}$, using 'control cells = UN ' as calibrator (arbitrary unit =1). Cell supernatants were also used for measurement (ELISA) of elafin and IL-8 protein levels.

Bottom panel : A549 cells were infected with live- or heat-inactivated (*)-PAO1 in MEM medium during either 2 or 6hrs. Cell supernatants and lysates were then recovered for assessment of RNA (elafin, IL-8, IFN-β) and protein levels (elafin, IL-8 by ELISA), respectively, as described above. Results are shown as means ± SD. Statistical significance: ANOVA, multiple comparison, Tukey's test, with each point representing an individual mouse, * : $p < 0.05$; **: $p<0.01$; *** :$p<0.001$; ****: $p<0.0001$).

Fig S2

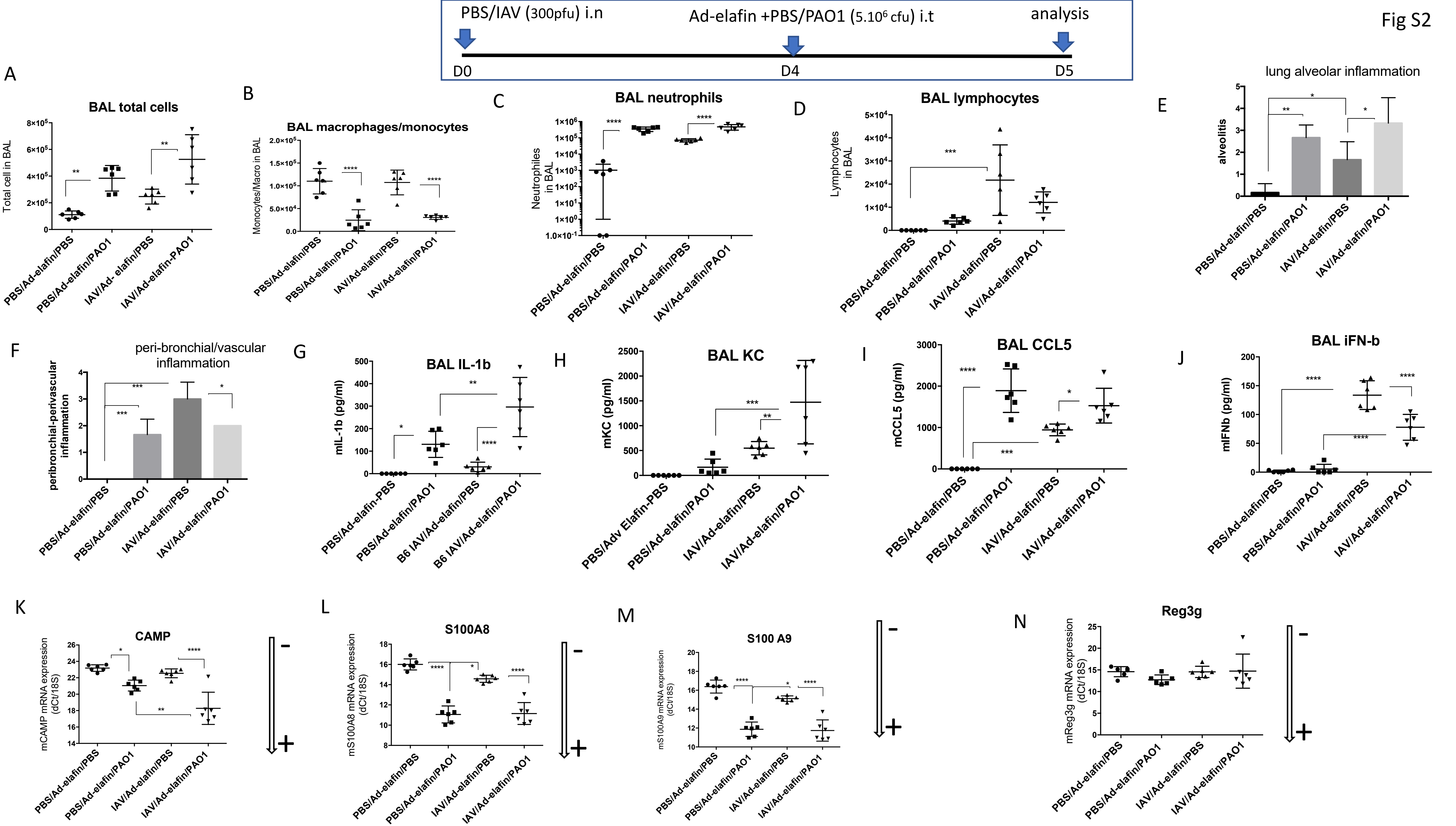
PBS/IAV (300pfu) i.n
Ad-elafin +PBS/PAO1 (5.10⁶ cfu) i.t
analysis
D0
D4
D5
A
BAL total cells
B
BAL macrophages/monocytes
C
BAL neutrophils
D
BAL lymphocytes
E
lung alveolar inflammation
F
peri-bronchial/vascular inflammation
G
BAL IL-1b
H
BAL KC
I
BAL CCL5
J
BAL iFN-b
K
CAMP
L
S100A8
M
S100 A9
N
Reg3g

Fig S2 IAV lung pre-infection sensitizes elafin-over-expressing mice (Ad-elafin) to further PAO1-mediated inflammation

BAL supernatants (see Fig 8 legend) were further used for the assessment of cellularity and lung inflammation (panels A-F), cytokine levels (panels G-J). Lung extracts were also used for RT-PCR analysis of antimicrobial molecules (panels K-N) and RNA expression was marked with an arrow indicating low (-) or high (+) level of expression). Results are shown as means ± SD. Statistical significance: ANOVA, multiple comparison, Tukey's test, with each point representing an individual mouse, * : $p < 0.05$; **: $p<0.01$; *** :$p<0.001$; ****: $p<0.0001$).

Fig S3

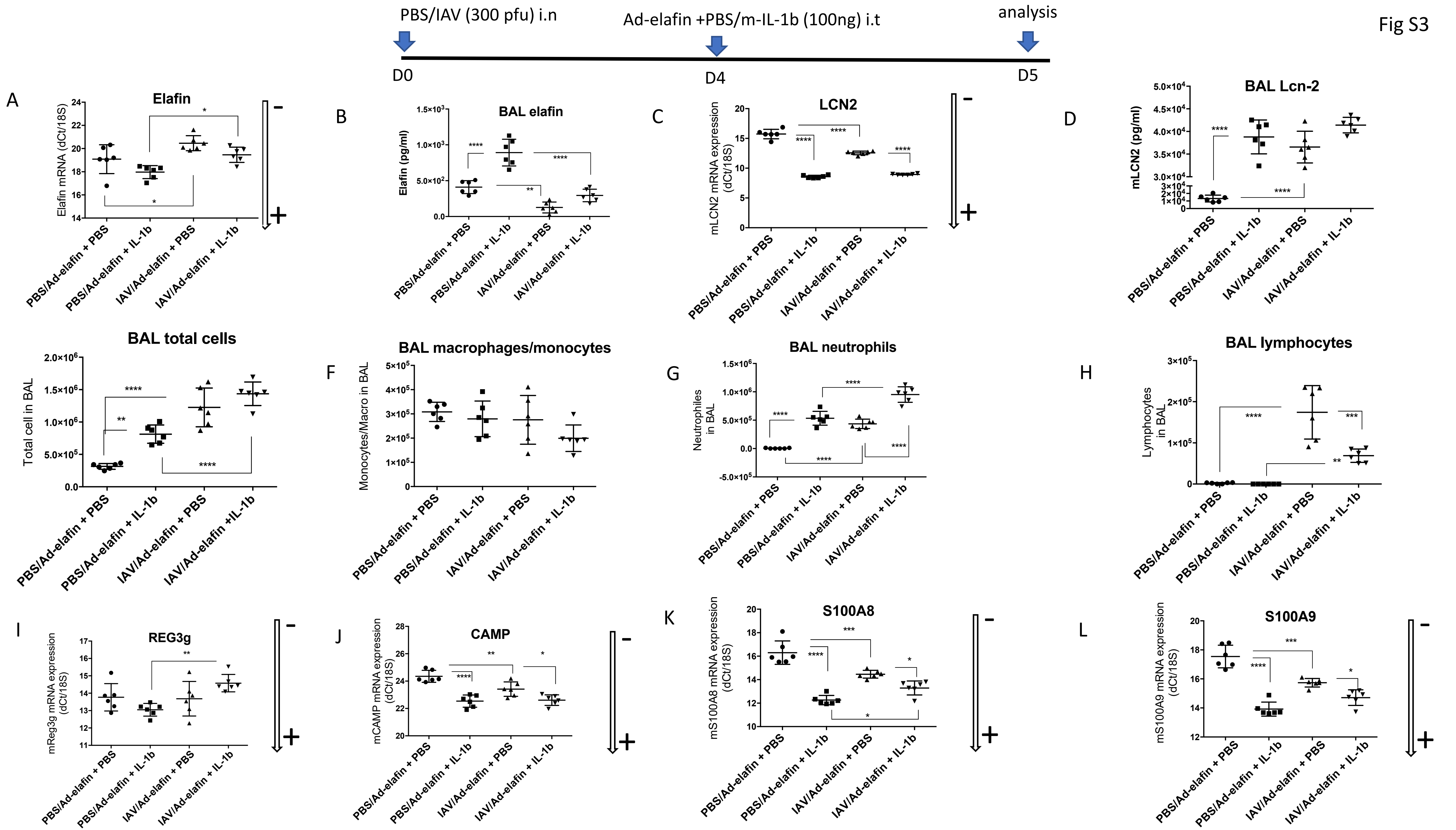

PBS/IAV (300 pfu) i.n
Ad-elafin +PBS/m-IL-1b (100ng) i.t
analysis
D0
D4
D5
A
Elafin
Elafin mRNA (dCt/18S)
B
BAL elafin
Elafin (pg/ml)
C
LCN2
mLCN2 mRNA expression (dCt/18S)
D
BAL Lcn-2
mLCN2 (pg/ml)
E
BAL total cells
Total cell in BAL
F
BAL macrophages/monocytes
Monocytes/Macro in BAL
G
BAL neutrophils
Neutrophiles in BAL
H
BAL lymphocytes
Lymphocytes in BAL
I
REG3g
mReg3g mRNA expression (dCt/18S)
J
CAMP
mCAMP mRNA expression (dCt/18S)
K
S100A8
mS100A8 mRNA expression (dCt/18S)
L
S100A9
mS100A9 mRNA expression (dCt/18S)
PBS/Ad-elafin + PBS
PBS/Ad-elafin + IL-1b
IAV/Ad-elafin + PBS
IAV/Ad-elafin + IL-1b
IAV/Ad-elafin +IL-1b

Fig S3 IAV lung pre-infection sensitizes mice to IL-1β-mediated cellular inflammation and down-regulates elafin protein accumulation

C57Bl/6 WT mice were instilled intra-nasally (i.n) with either PBS or IAV (300 pfu). 4 days later, mice were further instilled i.t with Ad-elafin (3.10^6 pfu) plus either PBS or IL-1β (100ng). At day 5, mice were sacrificed, and a bronchoalveolar lavage (BAL) was performed for cystospin cellular quantification (panels E-H), elafin and Lcn-2 protein content (panels B, D). Lungs were also obtained for q-PCR assessment of a variety of antimicrobials (panels I-L) and that of elafin and Lcn-2 (panels A, C) and RNA expression was marked with an arrow indicating low (-) or high (+) level of expression. Results are shown as means ± SD. Statistical significance: ANOVA, multiple comparison, Tukey's test, with each point representing an individual mouse, * : $p < 0.05$; **: $p<0.01$; *** :$p<0.001$; ****: $p<0.0001$).

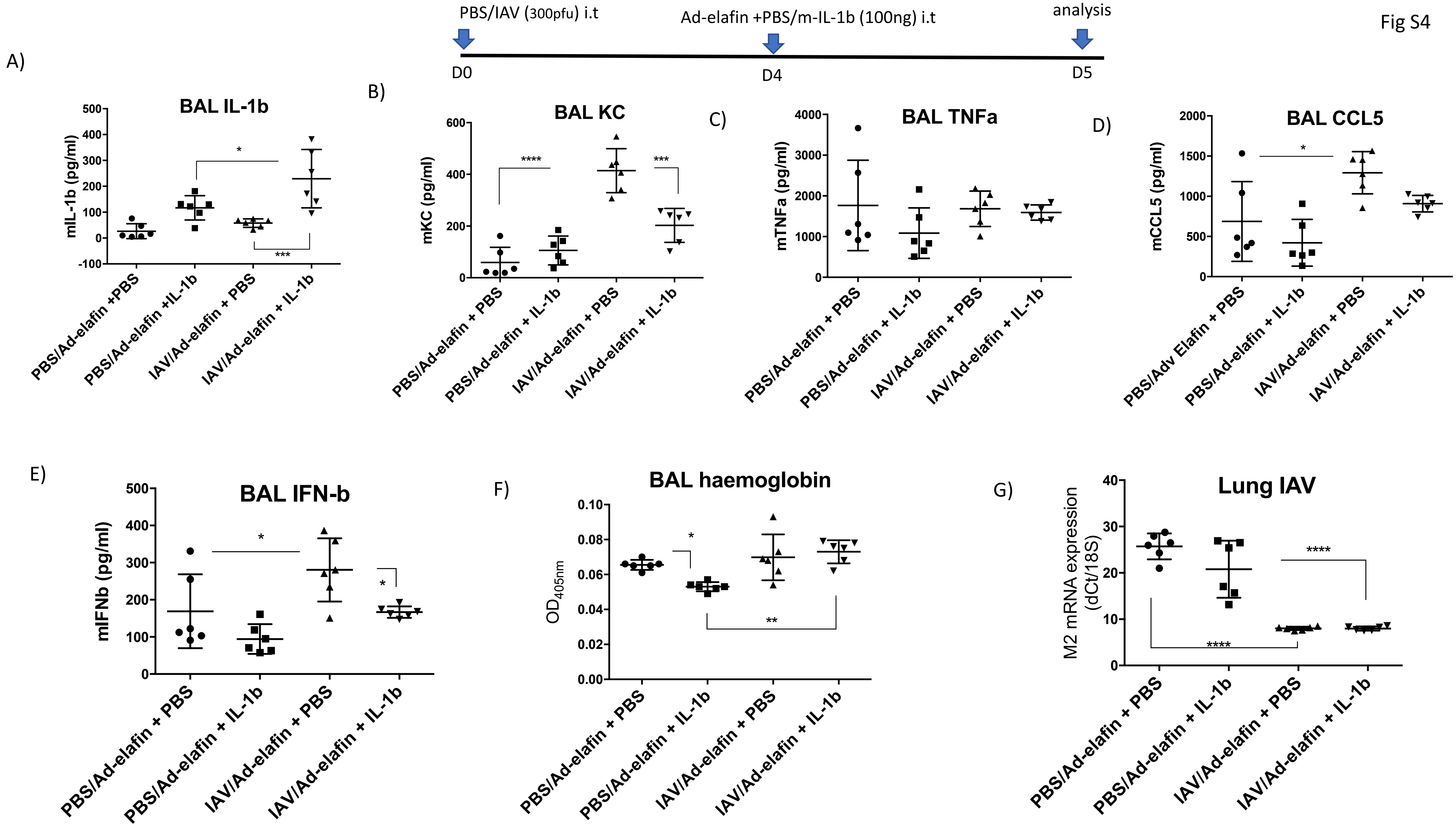
Fig S4
PBS/IAV (300pfu) i.t
Ad-elafin +PBS/m-IL-1b (100ng) i.t
analysis
D0
D4
D5
A)
BAL IL-1b
mIL-1b (pg/ml)
PBS/Ad-elafin +PBS
PBS/Ad-elafin +IL-1b
IAV/Ad-elafin + PBS
IAV/Ad-elafin + IL-1b
B)
BAL KC
mKC (pg/ml)
C)
BAL TNFa
mTNFa (pg/ml)
D)
BAL CCL5
mCCL5 (pg/ml)
PBS/Adv Elafin + PBS
E)
BAL IFN-b
mIFNb (pg/ml)
F)
BAL haemoglobin
OD405nm
G)
Lung IAV
M2 mRNA expression (dCt/18S)
PBS/Ad-elafin + PBS
PBS/Ad-elafin + IL-1b
IAV/Ad-elafin + PBS
IAV/Ad-elafin + IL-1b

Fig S4 IAV lung pre-infection sensitizes mice to IL-1β-mediated cytokine production

BAL supernatants (see Fig S3 legend) were further used for quantification of protein levels of cytokines (panels A-E), haemoglobin (panel F), and IAV RNA content (panel G). Results are shown as means ± SD. Statistical significance: ANOVA, multiple comparison, Tukey's test, with each point representing an individual mouse, * : $p < 0.05$; **: $p<0.01$; *** :$p<0.001$; ****: $p<0.0001$).
